# Supplementary material for: Analysis of the indispensable RAD51 cofactor BRCA2 in Naganishia liquefaciens, a Basidiomycota yeast
Source: Life Sci Alliance. 2023 Nov 28;7(2):e202302342. doi: 10.26508/lsa.202302342 (PMC10684384; doi:10.26508/lsa.202302342)
Supplement: Supplementary file 1 [file LSA-2023-02342_TableS1.docx]

**Table S1.** Taxonomical distribution of BRCA2 homologs.

| **A.** Eukaryota |  |  |
| --- | --- | --- |
| Taxonomy |  | Number of BRCA2 homologs |
| Opisthokonta | Fungi | 675 |
|  | Metazoa | 953 |
|  | Amoeboaphelidium | 2 |
|  | Capsaspora owczarzaki ATCC 30864 | 1 |
| Viridiplantae | | 269 |
| Discoba | | 5 |
| Amoebozoa | | 10 |
| Sar | | 55 |
| Metamonada | | 6 |
| Thecamonas trahens ATCC 50062 | | 1 |
| Pavlovales | | 2 |
| Rhodophyta | | 5 |
|  |  |  |
| **B.** Fungi |  |  |
| Taxonomy |  | Number of BRCA2 homologs |
| Basidiomycota | Agaricomycotina | 350 |
|  | Pucciniomycotina | 25 |
|  | Wallemia | 5 |
|  | Ustilaginomycotina | 42 |
| Fungi incertae sedis | Chytridiomycota incertae sedis | 58 |
|  | Mucoromycota | 110 |
|  | Zoopagomycota | 75 |
|  | Blastocladiomycota incertae sedis | 8 |
|  | Paramicrosporidium saccamoebae | 1 |
|  | Encephalitozoon cuniculi GB-M1 | 1 |
|  |  |  |
