# Supplementary material for: Analysis of the indispensable RAD51 cofactor BRCA2 in Naganishia liquefaciens, a Basidiomycota yeast
Source: Life Sci Alliance. 2023 Nov 28;7(2):e202302342. doi: 10.26508/lsa.202302342 (PMC10684384; doi:10.26508/lsa.202302342)
Supplement: Supplementary file 5 [file LSA-2023-02342_TableS2.docx]

**Table S2.** All strains are derivatives of the *N. liquefaciens* N6 strain.

| **Strain** | **Genotype** | **Source** |
| --- | --- | --- |
| MP1 | Wild type *N. liquefaciens* N6 | (*Abe* et al. 2006) |
| MP35 | *rad51::NAT* | (*Palihati* et al. 2021) |
| MP31 | *brh2::NAT* | This study |
| MP33 | *rad52::NAT* | (*Palihati* et al. 2021) |
| MP37 | *rad51::NAT brh2::NEO* | This study |
| MP39 | *rad51::NAT rad52::NEO* | (*Palihati* et al. 2021) |
| MP41 | *brh2::NEO rad52::NAT* | This study |
| MP101 | *rad51::NAT brh2::NEO rad52::HYG* | This study |
| MP7 | *dss1::NAT* | This study |
| MP94 | *ku70:: HYG* | This study |
| MP75 | *rad52::HYG ku70::NEO* | (*Palihati* et al. 2021) |
| MP108 | *rad51::NAT ku70::NAT* | (*Palihati* et al. 2021) |
| MP112 | *rad51::NAT rad52::HYG* | (*Palihati* et al. 2021) |
| MP113 | *rad51::NAT rad52::HYG ku70::NAT* | (*Palihati* et al. 2021) |
| MP127 | *brh2::NAT ku70:: HYG* | This study |
| MP131 | *rad51::NAT ku70::NAT brh2:: HYG* | This study |
| MP139 | *brh2::NAT rad52::HYG* | This study |
| MP143 | *brh2::NAT ku70::NAT* | This study |
| MP147 | *brh2::NAT rad52::HYG ku70::NAT* | This study |
| MP151 | *rad51::HYG brh2::NAT* | This study |
