# Supplementary material for: Analysis of the indispensable RAD51 cofactor BRCA2 in Naganishia liquefaciens, a Basidiomycota yeast
Source: Life Sci Alliance. 2023 Nov 28;7(2):e202302342. doi: 10.26508/lsa.202302342 (PMC10684384; doi:10.26508/lsa.202302342)
Supplement: Supplementary file 6 [file LSA-2023-02342_TableS3.docx]

**Table S3.** Primers used in this study

| **number** | **sequence** |
| --- | --- |
| Pr-111 | TGACCAAGGTGTTCCCCGAC |
| Pr-112 | TGACGTTGGTGACCTCCAGC |
| Pr-140 | TCGCTCACATCGCCTCCAGCTAAGCTTGAAGATCTACAGCAGCAGCAACCTCAACAACCTCGGCGAATTC |
| Pr-141 | CTGCTGTTCGTCGCGAAGTATATCATTTCGTTACATTTTGTCACATCTTGTTAGGGGCAGGGCATGCTCA |
| Pr-146 | ACGTGTATTACGCGTCGCTC |
| Pr-147 | AGCGTCAGCGCAATCATTGA |
| Pr-156 | ACCTCTAGAATGGCGTATGCAGTGCAGTG |
| Pr-157 | ACCCATATGTGGCGGACGGAATCGATGTA |
| Pr-158 | ACCCCATGGATTACAACAGGCTTTCCAGG |
| Pr-159 | ACCGGATCCTAACGGAGAGATCTCCTCCG |
| Pr-172 | AGAGAATCGCGTTGTGATCG |
| Pr-173 | ACAATCATTCGAGGCCCGTC |
| Pr-174 | ATTCCACTCTCGGTCTCGGT |
| Pr-175 | TCCGAAGATACGAGTCGCTG |
| Pr-320 | CATATGGGTTGGATCCCTTAATAGCATCACCCAGTATTCAAGAAAAAATCCAGACCGTTTGATGCTTCTAGGGAAACGGGAGACCGTGACGAGCATAACG |
| Pr-321 | AGTCAAGTGGATGTATACATGAAGAAGCGCCGGTCATACATCGATCATTCTAGATGCCGCGTGCATAGCGAGTTATCTGCCAGTATAGCGACCAGCATTC |
| Pr-342 | TTTCGTCTCTGTCGGTTTCTCCAC |
| Pr-343 | AAACGTGGAGAAACCGACAGAGAC |
| Pr-361 | TTTCGTACGGCGGTCATCACGGCA |
| Pr-362 | AAACTGCCGTGATGACCGCCGTAC |
| Pr-401 | CATATGGGTTGGATCCCTTAATAGCATCACCCAGTATTCAAGAAAAAATCCAGACCGTTTGATGCTTCTAGGGAAACGGGTCAACAACCTCGGCGAATTC |
| Pr-402 | AGTCAAGTGGATGTATACATGAAGAAGCGCCGGTCATACATCGATCATTCTAGATGCCGCGTGCATAGCGAGTTATCTGCCCCCATCCCCAATCTTTCTT |
| Pr-403 | GCATCTCGAAGCACATCACCACACCACCAGCCAGACAAACCTCCAAGAGAAAGCTCGGATCCACGTCTTCCAGTGTTTCCTCAACAACCTCGGCGAATTC |
| Pr-404 | GTATCATCCTACTCTACATTCACATTCATACGCGCGTCCCGTATCGTGTCTGTATGCACAAAGACCCGTGGCAGGACAGTCCCCATCCCCAATCTTTCTT |
| Pr-458 | AACCATATGATGGCGACCCAAGAATACGC |
| Pr-459 | AACGAATTCTTACTTGTCACCTTCGTCTTGCG |
| Pr-460 | AACCATATGATGGTATCGCACAGCACAGT |
| Pr-461 | AACGGATCCCTATTCTGGACAACGTTTCCATGT |
| Pr-504 | ACCTGAGAGCTGATCGATGG |
| Pr-505 | AGACACGCTTATCGGAATCG |
| Pr-550 | ACCTCTAGACATATGATGTCGACGCTGTCGGGACA |
| Pr-551 | ACCTCTAGACATATGTCATGCTTTGCTGCGTTTGG |
| Pr-567 | ACCCATATGATGGCAGCCCAACCACCA |
| Pr-568 | ACCGGATCCTTACATTTTCATCGCATCTGGATCGT |
| Pr-620 | ATGGCGTATGCAGTGCAGTGGTGTCCGCTTCATAAAGGTTGCATTGTGTCAATCAATAAAGATAGCTCAAGCCCGAGTACGTTACCGGATAAGGCGCAGC |
| Pr-621 | AGGAAGATGCAGCTGCTGATCAGGCTGCGATGGAAAAGCTCGACACAGACTCTGTTAACGGAGAGATCTCCTCCGCTGATCCAATAGGCCGAAATCGGCA |
| Pr-622 | ACCGGATCCGTTAACTTACTGGTTCAGCATGCCAT |
| Pr-623 | ACCGTTAACAGGTCTTCATTGTGTGAAGT |
